# *Nannochloropsis oceanica* as a Source of Bioactive Compounds: Mapping the Effects of Cultivation Conditions on Biomass Productivity and Composition Using Response Surface Methodology

**DOI:** 10.3390/md22110505

**Published:** 2024-11-06

**Authors:** Emil Gundersen, Jette Jakobsen, Susan Løvstad Holdt, Charlotte Jacobsen

**Affiliations:** National Food Institute, Technical University of Denmark, Kemitorvet Bygning 202, 2800 Kongens Lyngby, Denmark

**Keywords:** *Nannochloropsis oceanica*, light intensity, temperature, salinity, protein, omega-3 fatty acids, vitamin K

## Abstract

Microalga *Nannochloropsis oceanica* presents a promising source of high-value food ingredients such as protein, omega-3 fatty acids, and vitamins. To fully unlock its potential, a thorough understanding of how cultivation conditions affect both growth and the nutritional composition is required. Hence, this study aimed to test and model the effects of temperature, light intensity, and salinity on biomass productivity and the final contents of protein, eicosapentaenoic acid (EPA), and vitamin K_2_ using response surface methodology (RSM). The RSM experiment revealed that the highest temperature and light intensity tested favored biomass productivity and protein content. According to the generated models, the two responses peaked with 0.135 g DM·L^−1^·day^−1^ and 0.559 g·g^−1^ DM, respectively, at 27 °C and 300–350 µmol·m^−2^·s^−1^. In contrast, the contents of both EPA and menaquinone-4 (MK-4), the only detected K vitamer, were stimulated at the lowest tested temperature. Based on the generated models, the two responses peaked with 0.037 g·g^−1^ DM and 89.3 µg·g^−1^ DM, respectively, at 19 °C combined with 3.0% salinity (EPA) or 120 µmol·m^−2^·s^−1^ (MK-4). Although additional optima may exist beyond the tested conditions, these findings provide valuable information on *N. oceanica*’s cellular response to changes in key cultivation conditions. Furthermore, it shows that two-stage cultivation may be needed to fully unlock the potential of this microalga as a future source of valuable lipid ingredients.

## 1. Introduction

Microalgae production presents a promising and largely untapped technology that can be used for the production of food ingredients to support a growing world population. In many cases, microalgae production offers a more eco-efficient alternative to conventional food sources. The production can take place on non-arable land using non-potable water, with areal productivities higher than most plant crops [1,2]. In addition, microalgae can utilize different industrial side streams as sources of carbon and inorganic nutrients, further strengthening their environmental profile [3]. Microalgae contain many components that are valuable for human health. Apart from being rich in protein and essential amino acids, microalgae also produce polyunsaturated fatty acids (PUFAs) and vitamins such as vitamin A (as beta-carotene), B, C, and E [4,5]. Other compounds of interest include antioxidative pigments such as astaxanthin, which is already in use in the animal feed industry [5].

In recent years, there has been increasing interest, both scientific and commercial, in species from the *Nannochloropsis* genus, a group of small, primarily marine microalgae. The genus is well known for its ability to accumulate large amounts of lipids, including the omega-3 fatty acid eicosapentaenoic acid (EPA) [6,7]. An adequate intake of omega-3 fatty acids is essential for both visual function and neural development, and EPA is of considerable nutritional importance, as the human body has limited endogenous production [8,9]. The specific species *Nannochloropsis oceanica* is also characterized by a high production of EPA, which typically accounts for 3–6% of the DM content [10,11]. In addition to the high content of EPA, *N. oceanica* has a high protein content (40–50% of DM), containing all essential amino acids required by humans [12,13]. *N. oceanica* is also a rich source of vitamins, including vitamin K, an indispensable micronutrient that is crucial for our ability to perform blood clotting [14]. Recently, it has also been demonstrated that *N. oceanica*’s biomass contains menaquinones (vitamin K_2_), more specifically 27 µg·g^−1^ of menaquinone-4 (MK-4) [15]. This is a rather special feature, given that menaquinones are typically restricted to food products of either animal or bacterial origin [16]. Owing to this impressive nutritional profile, *N. oceanica* has the potential to become a complete source of non-animal protein, omega-3 fatty acids, and vitamin K_2_.

As with other species of microalgae, both the growth speed and chemical composition of *N. oceanica* can be modulated by use of different cultivation conditions. Commonly controlled elements include abiotic factors such as light intensity, temperature, and nutrients’ availability [17]. Especially abiotic stresses, such as high light stress or nitrogen deprivation, can be used to trigger the production of specific metabolites, including many valuable lipids [18]. This typically happens at the expense of growth, which is why these stress factors are often applied after the main growth phase in so-called two-stage cultivation [19]. Alternatively, more moderate changes in cultivation conditions can be applied continuously to balance biomass production and compound accumulation. In both cases, understanding and being able to predict the cellular response to these changes is an essential part of optimizing any microalgae production.

Several cultivation studies have already demonstrated how *N. oceanica* responds to changes in important abiotic factors [10,20,21,22,23,24,25,26]. Due to its oleaginous properties, many of these previous cultivation studies have mainly focused on the promotion of biomass and lipids, especially EPA, with the aim of enhancing the final productivity. For example, ref. [20] investigated how increasing light intensity under nitrogen replete conditions affected the accumulation of biomass and lipids in *N. oceanica* IMET1. Similarly, ref. [21] examined how the growth and fatty acid distribution changed in *N. oceanica* acclimated to different temperatures. A similar focus on growth and lipid production was also the case in [22], which worked with large-scale cultivation of *N. oceanica* in outdoor tubular photobioreactors (PBRs). As a result, the lipid metabolism of *N. oceanica* is relatively well-researched and understood. A few studies have also assessed cultivational changes in the protein content of *N. oceanica* [15,24] whilst no study, to our knowledge, has yet shown how the content of vitamin K is affected by abiotic factors such as light intensity and temperature. However, to fully unlock the potential of *N. oceanica* as a future source of food ingredients, nutritionally important compounds like protein and vitamins need to be considered in parallel with lipid production.

Therefore, this study aimed to do a more comprehensive evaluation of *N. oceanica*’s compositional response to changes in the key cultivation factors of light intensity, temperature, and salinity. To exclude stress-induced changes, the factors were tested within a non-stressful range under nutrient-replete conditions. Using a Box–Behnken design (BBD) with three numeric factors on three levels, *N. oceanica* cultures were exposed to thirteen different combinations of light intensity, temperature, and salinity. At a specific cell density (based on light absorbance), cultures were terminated, and the final dry-matter content used for an estimation of biomass productivity. Biomass was harvested from the remaining culture volume and used for determining the content of protein, EPA, and vitamin K_2_—in total, four cellular responses. Using response surface modeling, the combined effects of the tested cultivation factors on each response were modelled individually. The most important factors for each response were identified, and a precision test was performed to validate the models. In doing so, four predictive models were generated that together give a comprehensive understanding of how subtle changes in growth environment affect the productivity and nutritional value of *N. oceanica*’s biomass.

## 2. Results

For the response surface study, temperature, light intensity, and salinity were selected as independent factors for modelling, whilst all other cultivation factors were kept fixed. A combination of treatments at three levels was created, with four replicates of the central point to increase precision in the center of the design space. Biomass productivity and the final content of protein, EPA, and MK-4 were taken as the response factors (dependent variables). In total, 16 cultures were grown (in two rounds of eight) and the response factors analyzed for all experimental units. The obtained values were fitted into the polynomial equation (Equation (6)), and each response factor was modelled individually. The actual and predicted results from the response surface study are shown in Table 1.

### 2.1. Modelling Biomass Productivity

The actual biomass productivity varied from a minimum of 0.050 g DM·L^−1^·day^−1^ to a maximum of 0.141 g DM·L^−1^·day^−1^, corresponding to a 2.8-fold (180%) increase across the tested factor combinations. The generated model was significant, with an F-value of 33.9 and *p*-value *<* 0.001. The predicted R^2^ adj. value of 0.868 indicated that the generated model could account for 86.8% of the observed response variability. The model also had an insignificant (*p*-value 0.356) lack of fit, indicating overall acceptance and reliability of the model. The full statistical evaluation of the biomass productivity model can be found in Table A1. Only the linear terms for temperature (T) and light intensity (LI), as well as the quadratic term of light intensity (LI^2^), were significant in the model, indicating that temperature and light intensity were the most influential parameters for enhancing biomass productivity. The simplified polynomial equation after removing insignificant terms is shown in Equation (1):

Biomass productivity (g DM·L^−1^·day^−1^) = 0.111 + 0.012·T + 0.029·LI − 0.016·LI^2^ (1)

**Figure 1:**
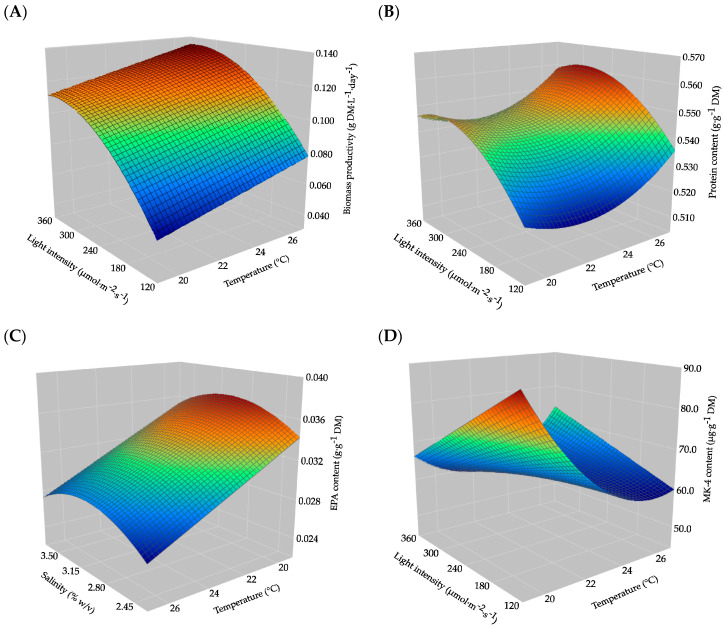
Three-dimensional response surface plots for generated models on (**A**) biomass productivity, (**B**) protein content, (**C**) eicosapentaenoic acid (EPA) content, and (**D**) menaquinone-4 (MK-4) content in *Nannochloropsis oceanica*. Dark blue represents the minimum response area and dark red the maximum response area. For visual reasons, the temperature scale on the graph for EPA content is inverted.

According to the simplified model, biomass productivity peaked at 27 °C and 350 µmol·m^−2^·s^−1^ and was unaffected by salinity within the tested ranges. The 3D response surface plot also showed that increasing temperature and light intensity increased biomass production. However, further increases in light intensity beyond 350 µmol·m^−2^·s^−1^ resulted in a small decrease in the productivity according to the model (Figure 1A).

### 2.2. Modelling Protein Content

The actual protein content varied from a minimum of 0.513 g·g^−1^ DM to a maximum of 0.566 g·g^−1^ DM, corresponding to a 1.1-fold (10%) increase across the tested factor combinations. The generated model was significant, with an F-value of 8.26 and *p*-value of 0.0025. The predicted R^2^ adj. value of 0.660 indicated that the generated model could account for 66.0% of the observed response variability. The model also had an insignificant (*p*-value 0.610) lack of fit, indicating overall acceptance of the model. The full statistical evaluation of the protein model can be found in Table A1. After removing insignificant terms, the linear and quadratic terms for temperature (T & T^2^) and light intensity (LI & LI^2^) were significant in the model, indicating that temperature and light intensity were the most influential parameters. The simplified polynomial Equation (2) is shown below:

Protein content (g·g^−1^ DM) = 0.543 + 0.006·T + 0.011·LI + 0.010·T^2^ − 0.012·LI^2^ (2)

According to the simplified model, protein content peaked at 27 °C and 300 µmol·m^−2^·s^−1^ and was unaffected by salinity within the tested ranges. The 3D response surface plot also showed that increasing the cultivation temperature enhanced the content, particularly beyond 22 °C. Increasing light intensity also enhanced the content, but further increases beyond 300 µmol·m^−2^·s^−1^ resulted in a decrease, as seen in the biomass productivity model (Figure 1B).

### 2.3. Modelling EPA Content

In the biomass samples, one of the most abundant fatty acids was EPA, representing 19–28% of the total fatty acid content. For EPA, the actual content on a dry-matter basis varied from a minimum of 0.025 g·g^−1^ DM to a maximum of 0.038 g·g^−1^ DM, corresponding to a 1.5-fold (52%) increase across the tested factor combinations. The generated model was significant, with an F-value of 11.2 and *p*-value of 0.0008. The predicted R^2^ adj. value of 0.672 indicated that the generated model could account for 67.2% of the observed response variability. However, after removing insignificant terms, the model had a significant (*p*-value 0.036) lack of fit, indicating that it did not accurately capture all the trends apparent in the data. The full statistical evaluation of the EPA model can be found in Table A1. Only the linear terms for temperature (T) and salinity (S), as well as the quadratic term of salinity (S^2^), were significant in the model, indicating that temperature and salinity were the most influential parameters for enhancing EPA content. The simplified polynomial Equation (3) is shown below:

EPA content (g·g^−1^ DM) = 0.033 − 0.004·T + 0.0005·S − 0.0024·S^2^ (3)

According to the simplified model, EPA content peaked at 19 °C and 3.0% (*w*/*v*) salinity and was unaffected by light intensity within the tested ranges. The 3D response surface plot also displayed that decreasing the cultivation temperature enhanced EPA content. Increasing salinity from 2.3% also enhanced EPA content; however, further increases beyond 3.0% resulted in a slight decrease according to the model (Figure 1C).

In addition to EPA, the most abundant fatty acids in the biomass samples were palmitic acid (C16:0) and palmitoleic acid (C16:1 n-7), representing in the range of 17–23% and 24–28% of the total fatty acid content, respectively. Table A2 shows the complete fatty acid composition for the biomass samples with the lowest measured EPA content (unit 1) and the highest measured EPA content (unit 13). The content of saturated fatty acids (SFAs), mainly palmitic acid, was 32.3% higher in biomass from unit 1, whilst the content of monounsaturated fatty acids (MUFAs), mainly palmitoleic acid, was 9.3% higher. In contrast, the overall content of polyunsaturated fatty acids (PUFAs), including EPA, was 25.1% higher in biomass from unit 13.

### 2.4. Modelling Content of MK-4

Of the eight vitamin K vitamers (PK, MK-4 to MK-10), MK-4 was the only vitamer present in the biomass, independent of cultivation treatment. The actual content varied from a minimum of 49.6 µg·g^−1^ DM to a maximum of 88.5 µg·g^−1^ DM, corresponding to a 1.8-fold (80%) increase across the tested combinations. After excluding unit 15 (obvious outlier) and removing insignificant terms, the model was significant, with an F-value of 5.88 and *p*-value of 0.0107. The model also had an insignificant (*p*-value 0.086) lack of fit, indicating overall acceptance of the model. The predicted R^2^ adj. value of 0.585 indicated that the generated model could account for 58.5% of the observed response variability. The full statistical evaluation of the MK-4 model can be found in Table A1. Both the linear terms for temperature (T) and light intensity (LI), as well as the quadratic term of temperature (T^2^) and the interactive term of temperature/light intensity (T·LI), were significant in the model, indicating that temperature and light intensity were the most influential parameters for enhancing MK-4 content. The simplified polynomial Equation (4) is shown below:

MK-4 content (µg·g^−1^ DM) = 61.74 − 5.39·T − 2.31·LI + 10.53·T^2^ + 9.28·T·LI (4)

According to the simplified model, MK-4 content peaked at 19 °C and 120 µmol·m^−2^·s^−1^ and was unaffected by salinity within the tested ranges. As also revealed by the 3D response surface plot, decreasing the cultivation temperature and light intensity enhanced MK-4 content. However, above a certain temperature (approx. 24 °C), both temperature and light intensity became positive, giving rise to a secondary peak (Figure 1D).

### 2.5. Evaluation of Model Precision

To evaluate the precision of the generated models, three validation units were subsequently cultivated with the combination of 27 °C, 350 µmol·m^−2^·s^−1^, and 3.0% salinity—the predicted optimum for biomass productivity. The mean response value was then compared with the predicted response range (mean ± 95% CI) of the different models. All responses for the validation units, except for the protein content, fell within the predicted response range (Table 2). The difference between means, that is the actual mean value for the validation units and the model-predicted means, varied from 7.1% (EPA) to 13.9% (MK-4).

## 3. Discussion

In this RSM study, the effects of the cultivation factors temperature, light intensity, and salinity on the growth and biochemical composition of *N. oceanica* were investigated. This study aimed to model these effects for cultures growing under overall replete conditions, excluding effects caused by abiotic stresses. Based on previous cultivation studies working with temperature [21,22,25], light intensity [20,24,27] and salinity [23,28,29], the non-stressful ranges were chosen to be 19–27 °C, 120–360 µmol·m^−2^·s^−1^, and 2.3–3.7% (*w*/*v*) salinity. Within these ranges, biomass productivity and the final content of protein, EPA, and vitamin K_2_ in harvested biomass were analyzed and modelled.

### 3.1. Biomass Productivity Is Dominated by Light Intensity

Biomass productivity is often the most crucial when optimizing the production of food ingredients using microalgae, since this dictates the yield of the product to sell and utilize. In this study, biomass productivity was also, by far, the response with the largest enhancement potential, increasing by 180% across the tested design space. Light intensity proved to be the most powerful factor regarding biomass productivity, with the linear term having almost three times the effect of temperature until reaching the optimum level. Several studies have reported similar findings on light intensity and biomass productivity. Cultivating *N. oceanica* CCALA 804 in a comparable setup at 25 °C and sparging with 2% CO_2_ [27] found increasing growth rates when gradually amplifying light intensity from 50 to 300 µmol·m^−2^·s^−1^. This tendency was also observed in [20], which obtained the highest dry-cell-weight accumulation (34.14 mg·L^−1^·h^−1^) when cultivating *N. oceanica* IMET1 at 331 µmol·m^−2^·s^−1^ compared to 71 and 161 µmol·m^−2^·s^−1^. This biomass productivity is approximately 6-fold higher than the predicted peak productivity found in this study at 350 µmol·m^−2^·s^−1^. However, this value was measured for a steady-state, continuous culture of *N. oceanica* [20]. However, comparison between studies on light intensity can be challenging as the optimal level depends on temperature, nutrient status, and preculture conditions [6]. In addition, the procedure of measuring and reporting light intensity often varies from study to study. According to the generated model, in this present study, the positive effect of light intensity stagnates and becomes slightly negative after 350 µmol·m^−2^·s^−1^, indicating that the light saturation point for the culture has been reached. Again, this is likely to be setup specific, as other studies have shown that different species of *Nannochloropsis* can grow well at 700 µmol·m^−2^·s^−1^ [28] and even tolerate 1600 µmol·m^−2^·s^−1^ for 24 h [27].

Regarding the effect of temperature on biomass productivity, the results aligned with previous findings. Working in laboratory scale, such as 50 mL Erlenmeyer flasks, [25] showed that the growth rate of *N. oceanica* CCAP 849/10 increased with rising temperature up to approximately 30 °C, whereafter growth was negatively impacted. Another study also found optimal growth at 25–29 °C when cultivating *N. oceanica* in a 1.8 L photobioreactor under similar conditions, while growth stagnated at temperatures higher than 31 °C [21]. Furthermore, [22] demonstrated high biomass productivity when cultivating *N. oceanica* at 28 °C in outdoor tubular photobioreactors. Combined with the results from this study, the ideal growth temperature for *N. oceanica* seems to be in the range of 25–30 °C.

Salinity within the tested range proved to be insignificant for biomass productivity. In a similar RSM study [26], a Plackett–Burman design was used to screen cultivation factors for a significant effect on *N. oceanica* biomass production. A salinity range of 1.5–4.5% was tested and showed no significant effect on biomass production. In contrast, another study found a 12% reduction in the biomass accumulation rate from 0.76 g DM·L^−1^·day^−1^ in medium with 27 g·L^−1^ NaCl (2.7% *w*/*v*) to 0.64 g DM·L^−1^·day^−1^ in medium containing 40 g·L^−1^ NaCl (4% *w*/*v*) [29]. In general, *Nannochloropsis oceanica* is considered to have a wide growth-permissive range of salinity and can even be adapted (through many generations) to growing in freshwater [30].

### 3.2. Protein Content Remained Stable Across the Design Space

Several species of microalgae are considered promising future sources of non-animal protein due to their superior areal productivity and high protein content [2,31]. In this cultivation study, run with nutrient-replete conditions, the protein content of *N. oceanica* based on total nitrogen was calculated to 0.513–0.567 g·g^−1^ DM (51–57% of DM). This overall level is comparable with other studies performed under nutrient-replete conditions that found the protein content in *N. oceanica* biomass to be 47.7% [12], 49.6–54.9% [32], and 51.3% [13]. The results also showed that protein content increased with higher temperature and light intensity, peaking at 27 °C and 300 µmol·m^−2^·s^−1^ according to the generated model. However, the model failed to predict the mean content of the subsequent validation cultures (Table 2), despite passing the statistical evaluation. It is therefore questionable whether the model can be used for reliably predicting the exact protein content.

As *Nannochloropsis* has primarily been researched for its production of omega-3 fatty acids, there are limited cultivation studies focusing on changes in protein content. In contrast to the findings of this study, [24] found increasing light intensity (from 50 to 500 µmol·m^−2^·s^−1^) to have a negative effect on protein content in *N. oceanica* IMET1. This decrease in total protein was largely caused by downregulation of thylakoid proteins involved in the photosynthetic system. However, the culture was acclimatized to only 50 µmol·m^−2^·s^−1^ before exposure to different light intensities, whereas in this study, precultures were adapted to 240 µmol·m^−2^·s^−1^ before inoculating the RSM units. This key difference could explain the conflicting response for the cultures cultivated at high light intensity (360 µmol·m^−2^·s^−1^). Furthermore, a previous study also found salinity to have a significant effect on the protein content of *Nannochloropsis* sp., with the content being approximately 20% higher at 3.0% (*w*/*v*) compared to 2.0% and 4.0% (*w*/*v*) salinity [23]. This was demonstrated for a local strain isolated from the South China Sea, which may not be directly comparable to the acquired collection strain (*N. oceanica* NIVA-2/03) used in the present study. In the closely related species *Nannochloropsis gaditana*, [33] protein content was likewise found to be significantly influenced by cultivation temperature, with the effect depending on applied light intensity. In the range of 20–30 °C, the protein content remained relatively stable, varying a maximum of 4.3% when cultivated using 250 µmol·m^−2^·s^−1^ and 14.2% when using 500 µmol·m^−2^·s^−1^. Similarly, in our study, the maximum difference in protein content across the entire design space was only around 10%. This indicates that *N. oceanica* under nutrient-replete conditions can maintain a relatively stable protein content despite changes in these important abiotic factors.

It is also important to mention that the used analysis of total nitrogen may not be the most precise method for creating a model on protein content. Although the applied nitrogen-to-protein conversion factor (4.78) was developed specifically for marine microalgae, considering variations in non-protein nitrogen (NPN) across growth phases [34], it may not fully account for changes in the level of NPN caused by the different cultivation treatments. To make the model more specific, protein content could be calculated based on analysis of total amino acids [35]. This would further enable the modeling of individual amino acids of particular interest, for example, essential amino acids.

### 3.3. EPA Content Enhanced by Lower Cultivation Temperature

*Nannochloropsis oceanica* has been extensively researched for its lipid production, in particular, its ability to accumulate high amounts of the polyunsaturated, omega-3 fatty acid EPA [10,21,36,37]. An adequate intake of EPA and other omega-3 fatty acids is essential for human neural and eye development and strengthens the body’s anti-inflammatory processes [8,9]. As most dietary EPA is sourced from fish and seafood products, *N. oceanica* presents a promising non-animal source of this valuable compound. In this study, the EPA content of *N. oceanica* was measured to be in the range of 0.025 to 0.038 g·g^−1^ DM (2.5–3.8% of DM). This overall level is in line with previously reported contents of EPA, ranging from approximately 2.4 to 5.2% of the DM, when grown under nitrogen-replete conditions [10,36,38]. Despite being highly significant, the generated model for EPA had a significant lack of fit (*p* = 0.036), which indicates that some trends in the data are not fully incorporated in the model. This could be resolved by including the insignificant quadratic term of temperature (T^2^). In both cases, model predictions remained almost identical, and the model presented herein succeeded in predicting the mean EPA content of the subsequent validation cultures (Table 2).

The model also showed that EPA content was favored by low temperature and medium salinity, peaking at 19 °C and 3.0% (*w*/*w*), with temperature being the most impactful factor. This was as expected, as temperature is a well-known modulator of lipid metabolism in microalgae. Furthermore, a decrease in cultivation temperature typically leads to an increase in the level of unsaturated fatty acids, particularly to increase membrane fluidity [17,18]. This upregulation of unsaturated fatty acids has also been observed specifically for *N. oceanica*. In [10], an increase in EPA content from 3.1 g·g^−1^ DM to 5.0 g·g^−1^ DM was observed across a temperature range of 30–15 °C when using a flat-panel PBR (1.8 L cultures) and a similar medium based on Nutribloom. Similarly, ref. [21] found EPA content to peak at approx. 3.7 g·g^−1^ DM at 17 °C. However, the EPA content remained relatively stable, dropping only to 2.9 g·g^−1^ DM for biomass produced at 31 °C. In both studies, the lowered cultivation temperature also resulted in reduced growth rates, which significantly affected the final lipid productivity.

In this study, salinity was also a significant factor for EPA content, with a small, negative effect on both sides of 3.0% (*w*/*v*). A similar observation was made in the closely related *N. gaditana* [37]. Here, the level of EPA in the biomass (% of total fatty acids) peaked at a salt content of 30–35 g·L^−1^ (3.0–3.5% *w*/*v*) and dropped significantly when increased to 40 g·L^−1^ (4.0% *w*/*v*). Likewise, [28] demonstrated that the EPA content in *Nannochloropsis* sp. UTEX2379 was slightly higher (3.6% of DM) at 27 g NaCl·L^−1^ compared to 40 g NaCl·L^−1^ (3.3% of DM). However, the content increased even further to 4.3% of the DM when salinity was reduced to 13 g NaCl·L^−1^, a general trend also observed for *N. oceanica* and *N. oculata* [29,39].

Light intensity within the rested range was found insignificant for the final content of EPA. This contrasts with other studies, which have demonstrated a strong, negative effect of increasing light intensity [28,40]. However, as with the protein content, the lack of response to high light intensity may be a result of the experimental preculture being grown at a relatively high light intensity (240 µmol·m^−2^·s^−1^) compared to other studies. It would be interesting to further study this potential light adaptation, as high light intensity was the main factor driving biomass productivity, which in turn will determine the final EPA productivity of *N. oceanica*.

The total fatty acid analysis also revealed parallel changes in other fatty acids depending on the applied cultivation conditions. As an example, the complete fatty acid composition of unit 1 (lowest EPA content) and unit 13 (highest EPA content) was compared (Table A2). This revealed an overall higher level of SFAs, mainly C16:0, and MUFAs, mainly C16:1 (n-7), in the biomass from unit 1. As both light intensity and salinity were the same for these units, the increased content of especially SFAs must have been a result of the higher temperature. High cultivation temperatures have previously been shown to favor the formation of saturated fatty acids in many marine microalgae [41]. Again, this effect is strongly linked to the cellular control of membrane fluidity [42]. This specific response to elevated temperatures has also been observed for *N. oceanica*. In one study, the level of SFAs in the triacylglycerol (TAG) fraction increased from 43% at 17 °C to 56% at 29 °C, with C16:0 being the FA that changed the most [21]. A similar increase in SFAs, especially C16:0, was also observed in [22] when changing the cultivation temperature in outdoor tubular PBRs from 18 °C to 28 °C. The changes observed in the current study are therefore in line with previous findings within the field.

### 3.4. MK-4 Increased Under Low Temperature and Low Light Intensity

The RSM experiment revealed that the content of MK-4, which was the only detectable vitamin K vitamer in *N. oceanica,* varied between 49.6 µg·g^−1^ DM and 88.5 µg·g^−1^ DM. In our former study of *N. oceanica* in laboratory scale, we found a content of 27 µg MK-4·g^−1^ DM and <10 ng phylloquinone·g^−1^ DM [15]. The results from the present study therefore confirm the near absence of phylloquinone and the presence of MK-4 as the major vitamin K vitamer in *N. oceanica*’s biomass. Compared to the other responses, there was a relatively large spread in the MK-4 content for the four central units, with approximately 24% difference between the lowest and highest measured value. As mentioned in the method description, the precision of the MK-4 determination in *N. oceanica* has previously been established to be ±4.2% (based on eight technical replicates). This large spread must therefore have been largely caused by biological variation.

The content of MK-4 was enhanced by low temperature and low light intensity, peaking at 19 °C and 120 µmol·m^−2^·s^−1^ according to the generated model. A similar trend in the effect of light intensity on the content of vitamin K_1_ (phylloquinone) was observed by [43] in the cyanobacterium *Anabaena cylindrica*. As phylloquinone serves as an electron carrier for photosystem I (PSI) in plants, green algae, and some cyanobacteria [44], the authors hypothesized that the decrease in phylloquinone at high light intensity (320 µmol·m^−2^·s^−1^) was due to a reduction in the PSI/PSII ratio [43]. As both the present study and [15] measured minuscule amounts of phylloquinone in *N. oceanica*, it is possible that MK-4 serves the function as electron carrier for PSI in this microalga. This role of MK-4 has also been demonstrated for the cyanobacterium *Gloeobacter violaceus* [45] as well as for the red microalga *Cyanidium caldarium* [46]. The observed response to light intensity in *N. oceanica* may therefore be linked to adaptation of the photosystem stoichiometry.

Lower cultivation temperature also strongly favored the synthesis of MK-4, especially in combination with low light intensity. The effect of temperature on vitamin production in microalgae is poorly investigated, and no available information exists on its interplay with vitamin K_2_ synthesis. However, it is well documented that low (suboptimal) temperatures can cause an imbalance between energy input and consumption in photosynthetic organisms, in turn leading to changes in the photosynthetic apparatus [47]. Furthermore, it has been demonstrated that the green microalga *Chlorella vulgaris* performs structural adjustments, especially to its photosystems, in response to this low temperature imbalance [48]. If MK-4 does indeed serve as an electron carrier for PSI, this would explain why temperature also has a strong effect in *N. oceanica*. To fully establish the role of MK-4, the vitamin K analysis should be complemented with a more comprehensive study into the composition of the photosynthetic system under different cultivation conditions. Nonetheless, the findings presented herein validate *N. oceanica* as a rich source of vitamin K_2_. At present, there is no broad consensus as to whether vitamin K_2_, including MK-4, is nutritionally superior to vitamin K_1_. In addition, authorities such as the European Food Safety Authority (EFSA) do not distinguish between the different vitamers [49]. Nonetheless, considering the adequate daily intake set at 70 µg vitamin K·day^−1^ [49], the measured MK-4 content of 49.6 µg·g^−1^ DM to 88.5 µg·g^−1^ DM makes freeze-dried *N. oceanica* powder a potential ingredient for food enrichment or supplements.

### 3.5. Balancing Biomass Production and Nutritional Quality

Understanding how cultivation factors affect an organism’s ability to grow and synthesize selected compounds is a key requirement for any biological production process. In this study, several models were made that display how biomass productivity and the content of protein, EPA, and MK-4 in *N. oceanica* respond in relation to changes in light intensity, temperature, and salinity. Within the tested range, the models also identified an optimal combination of these factors in relation to each response. Ideally, the predicted optimum for each response should have been validated separately. However, due to time constraints, a common set of validation units cultivated at the predicted optimum for biomass productivity (27 °C + 350 µmol·m^−2^·s^−1^) was used to test the predictive precision of all models. As salinity within the tested range proved insignificant for biomass productivity, the optimal salinity for EPA content (3.0% *w*/*v*) was chosen as the third factor level. Validating biomass productivity was prioritized, as it is often the most crucial in microalgae production, usually outweighing any associated reduction in compound concentration caused by unfavorable cultivation conditions [21,43].

The obtained results and generated models clearly displayed a tradeoff between biomass production and nutritional quality. Under conditions favoring biomass accumulation and protein content, the final content of MK-4 and particularly EPA were at a suboptimal level (Figure 1). One solution to this could be the use of two-stage cultivation. In two-stage cultivation, the cultivation process is split in two phases, with conditions in the first phase typically favoring rapid cell growth and conditions in the second phase inducing accumulation of a specific compound [19]. Two-stage cultivation has been tested on several microalgal species for the production of various compounds. Examples include the production of astaxanthin by *Haematococcus pluvialis* induced by high light intensity in the second stage [50], the production of carotenoids in *Dunaliella salina* induced by a combination of low cell density and nitrogen limitation in the second stage [51], and the production of total lipids in *Scenedesmus obtusus* through increased salinity (salt stress) in the second stage [52]. Two-stage cultivation has also previously been applied to *Nannochloropsis* sp. for the enhanced production of EPA. In [53], the content of EPA (% of total lipids) was increased by 3.4-fold when subjecting a mature (late log phase) culture to low light (30 µmol·m^−2^·s^−1^) and low temperature (10 °C) for three days in both a photobioreactor (5 L) and flask setup (3 L). This concept could also be adapted to less extreme settings, like the ones tested in the current study. This would entail a primary growth phase at 27 °C and 360 µmol·m^−2^·s^−1^ and a secondary induction phase at 19 °C and 120 µmol·m^−2^·s^−1^. As revealed by the RSM experiment, such an approach would most likely also induce vitamin K_2_ (MK-4) content whilst having only a minor negative effect on the protein content. This kind of cultivation strategy should also be possible at a larger scale, since parameters such as cultivation temperature and light intensity are often controllable, at least in indoor PBR systems. The optimal parameter values are likely to be different in other systems, but the overall observed trends, such as the effect of lower cultivation temperature, should still apply at larger scale. If thoroughly researched and implemented, this kind of cultivation strategy could provide a powerful contribution to developing *Nannochloropsis oceanica* as a future source of these valuable food ingredients.

## 4. Materials and Methods

### 4.1. Microalgae Strain, General Maintenance & Precultures

*Nannochloropsis oceanica* (NIVA-2/03) from the Norwegian Culture Collection of Algae (Oslo, Norway) was initially decontaminated according to [54]. In brief, this was performed by restreaking twice on a medium containing ampicillin, cefotaxime, and carbendazim at concentrations of 700 µg·mL^−1^, 200 µg·mL^−1^, and 0.1 µg·mL^−1^, respectively. The purified culture was maintained on plates with an artificial sea water (ASW) medium consisting of 30 g·L^−1^ (3% *w*/*v*) sea salt (Instant Ocean, Blacksburg, VA, USA), 20 mM HEPES buffer at pH 7.5, 1% (*w*/*v*) agar, and 4 mL·L^−1^ Nutribloom Plus (Necton, Olhão, Portugal). This medium without agar is referred to as the standard medium. Plates were kept at 23 °C and illuminated with 150 µmol·m^−2^·s^−1^ continuous light. These plates were used to inoculate liquid precultures in a standard medium. Precultures were grown in 250 mL GLS80 bottles with a 4-port lid (Duran, Duisburg, Germany) at 23 °C and with 80 mL·min^−1^ sparging with atmospheric air. Approximately 240 µmol photons·m^−2^·s^−1^ continuous white light was applied to the cultures using T8 LED tubes (Systion Electronics, Palvilhão, Portugal). Precultures were cultivated until reaching a density sufficient for inoculating the main experimental units.

### 4.2. Design of Experiment (Box–Behnken Setup)

Cultivation of *N. oceanica* was evaluated using response surface methodology (RSM). A Box–Behnken experimental design (BBD) with three numeric factors on three levels was applied. This design consisted of 16 randomized experimental units with four replicates at the central point to minimize error. Ideally, all experimental units should have been run simultaneously, but due to technical limitations, the cultivation units were run in two independent rounds of eight units each. Temperature (°C), light intensity (µmol·m^−2^·s^−1^), and salinity (% *w*/*v*) were chosen as the independent factors. The minimum and maximum values were chosen based on literary research and represent a low and high value within a non-stressful range for cultivation of *N. oceanica*. All factors were coded according to Equation (5):

(5) $$X=\frac{X_{i}-X_{0}}{\Delta X}$$

where *X* is the coded value, *X_i_* is the corresponding actual value, *X*_0_ is the actual value in the center of the domain, and *ΔX* is the increment of *X_i_* corresponding to a variation of 1 unit of *X*. The actual and coded values for the cultivation factors are shown in Table 3.

Biomass productivity (g DM·L^−1^·day^−1^) and the content of protein (g·g^−1^ DM), EPA (g·g^−1^ DM), and MK-4 (µg·g^−1^ DM) were selected as the responses for the combination of the independent variables. The response variables were fitted to the following second-order polynomial model (Equation (6)) which, in general, can describe relationship between the responses and the independent variables [55]:

(6) $$Y=\beta_{0}+\sum_{i=0}^{3\;}\beta_{i}X_{i}+\sum_{i=0}^{3}\beta_{ii}X_{i}+\sum \sum_{i<j=1}^{3}\beta_{ii}X_{i}X_{j}$$

where *Y* represents the response variable, *X_i_* and *X_j_* are the independent factors affecting the response, and β_0_, β_i_, β_ii_, and β_ij_ are the regression coefficients for intercept, linear, quadratic and interaction terms. Each response was analyzed independently, and selection of the optimal conditions was based on desirability function. The experimental design and multiple linear regression analysis were performed using the software JMP Pro 16 (SAS Institute, Cary, NC, USA). Model precision was subsequently evaluated by cultivating three validation units at the predicted optimum for biomass productivity and comparing the mean response values with the predicted response range of the different models. The model prediction was considered precise if the mean response value of the validation units fell within the 95% CI of the predicted response mean.

### 4.3. Cultivation and Monitoring of Experimental Units

The main experimental units for the BBD setup, including the three validation units, were cultivated in a Fitoclima 600 growth chamber (Aralab, Rio de Mouro, Portugal) using 1000 mL GLS80 bottles with a 4-port lid (Duran, Duisburg, Germany). All flask cultures were set up with 800 mL total volume, a start ABS_750_ of approx. 0.100, and 100 mL·min^−1^ sparging with a 1.0% CO_2_ gas mixture. The standard medium was used, but with differing salinity. The cultures were then grown under selected combinations of temperature and light intensity according to the BBD setup; see Table 1. As for the precultures, continuous white light was applied to the cultures using T8 LED tubes (Systion Electronics, Palvilhão, Portugal). Light intensity was measured on the outside of the bottle (bottom, center) using a Universal Light Meter ULM-500 (Heinz Walz GmbH, Effeltrich, Germany). Growth was monitored daily by measuring absorbance at 750 nm using a Jenway 7205 UV/VIS spectrophotometer (Cole-Parmer, Vernon Hills, IL, USA). When above ABS_750_ 1.0, the absorbance measurements were performed on a diluted culture volume (ABS_750_ < 0.9). All cultures were harvested as soon as possible after reaching an ABS_750_ value of 6.0 (all within ABS_750_ 6.0–7.0).

### 4.4. Culture Dry Matter and Biomass Productivity

Dry matter (DM) content was determined using an in-house protocol suitable for use on marine microalgae. In brief, 10 mL of culture was sampled at the time of harvest and centrifuged at 8000× *g* for 10 min, 5 °C. The cell pellet was resuspended in 30 mL 0.5 M ammonium formate and centrifuged again at 8000× *g* for 10 min, 5 °C. The “washed” cell pellet was resuspended in 5 mL deionized H_2_O and transferred quantitatively to dried and preweighed beakers. The beakers were dried overnight at 95 °C, cooled to room temperature in a desiccator, and weighed again. DM content (g·L^−1^) was calculated as the weight difference divided by the used volume of algal suspension. Biomass productivity (net volume productivity) was then calculated as the final DM content divided by the time of cultivation in days until harvest (g DM·L^−1^·day^−1^). The DM analysis was carried out in technical duplicates and the mean used as input in the BBD. The used cultivation time and final DM content for all units can be found in supplementary Table A3.

### 4.5. Harvesting and Drying of Biomass

The remaining culture volume was harvested by centrifugation at 7000× *g* for 15 min at 5 °C. The pellet was resuspended in 400 mL 0.5 M ammonium formate and centrifuged again at 7000× *g* for 15 min at 5 °C. Pellets were resuspended in 10 mL deionized H_2_O and frozen down in freeze-drying flasks at −80 °C. Frozen samples were then freeze-dried for 48 h at 0.5 mbar and room temperature using a Telstar LyoQuest-55 (Azbil Co., Ltd., Tokyo, Japan). The freeze-dried biomass was pulverized using a porcelain mortar and transferred to airtight containers. To minimize degradation and loss of compounds, especially EPA and vitamin K, all powder samples were stored in the dark at −20 °C and analyzed within a few months.

### 4.6. Powder Moisture Content

Moisture content in powder samples was determined using standard methodology. Approximately 30 mg powder was transferred to a dried and preweighed beaker. The sample was dried overnight at 105 °C in a Dry-line convection oven (VWR, Radnor, PA, USA), cooled to room temperature in a desiccator, and weighed again. The sample weight before and after drying was then used to calculate the percentage of remaining moisture. The analysis was carried out as a single measurement, and the percentage of moisture was used to convert the content of protein, EPA, and MK-4 to a dry matter basis.

### 4.7. Analysis of Protein

Total nitrogen was determined using a Vario EL cube (Elementar, Langenselbold, Germany) according to the manufacturer’s instructions for CHNS analysis. Nitrogen measurements were carried out using a standard curve based on sulfanilamide and casein, having 16.7% N as internal control. Total nitrogen was then converted to protein using a conversion factor of 4.78, specific for marine microalgae [34]. The analysis was performed as a duplicate determination with a maximum allowed difference of 5% between replicates. The mean value of the two replicate measurements was used as input in the BBD.

### 4.8. Fatty Acid Analysis (Direct-FAME)

The fatty acid composition was determined using a two-step, direct transesterification of fatty acid methyl esters (direct-FAME) based on the work presented in [56]. Approximately 40 mg of freeze-dried powder was used per sample. First, 1 mL 1 M NaOH, 1 mL Toluene, and 200 µL internal standard (2% *w*/*v* C23:0 in n-heptane) were added to each sample. All glycerol-bound fatty acids were transesterified by incubating the samples in an ultrasound bath for 10 min, followed by a 100 °C water bath for 2 min. All free fatty acids were then methylated by adding 2 mL 20% bortriflourid in methanol, followed by a 100 °C water bath for 2 min. The samples were cooled down and 2 mL saturated NaCl solution and 1 mL n-heptane with 0.01% BHT added. The heptane phase, containing the fatty acid methyl esters, was transferred into GC vials and analyzed by gas chromatography combined with a flame ionization detector (GC-FID) (HP-5890 A, Agilent Technologies, Santa Clara, CA, USA). Separation was performed using an Agilent DB wax 127–7012 (10 μm × 100 μm × 0.1 μm) GC column (Agilent Technologies, Santa Clara, CA, USA). A gradient temperature program was applied (start 160 °C, 0.3 min at 200 °C, 4 min at 220 °C, 3.8 min at 240 °C), running a total of 15.7 min per sample. A standard mix of fatty acid methyl esters (Sigma, St. Louis, MO, USA) was used for fatty acid identification. The analysis was performed as a duplicate determination with a maximum allowed difference of 10% between replicates. The mean value of the two replicate measurements was used as input in the BBD.

### 4.9. Vitamin K Analysis

The vitamin K was extracted by enzymatic treatment and clean-up by liquid-liquid and solid-phase extraction for final detection and quantification by LC-MS/MS, as previously described in detail in [16]. Briefly, 10 mg freeze-dried sample was weighed in a 15 mL Sarstedt tube, followed by addition of 125 ng deuterium labelled (*d*7) of PK and MK-4, MK-7, and MK-9 (IsoScience LLC, Ambler, PA, USA) as internal standards. The extraction process included heating in 2-propanol, lipid degradation using the two lipases, Lipozyme^®^ TL 100 L and LecitaseTM Ultra (Novozymes A/S, Bagsværd, Denmark), clean-up by liquid-liquid extraction (2-propanol and *n*-heptane), and silica solid-phase extraction (Biotage, Uppsala, Sweeden) by elution with *n*-heptane and ethyl acetate. Final detection was performed by a UHPLC-MS/MS (1290 Infinity II and 6470 triple quadrupole mass spectrometer, Agilent Technologies, Santa Clara, CA equipped with a Ascentis^®^ Express C18 column (10 cm × 2.1 mm, 2 µm and a guard column, Supelco, Bellafonte, PA, USA). The precision of MK-4 assessed in a house-reference sample (also *N. oceanica*) was 21.7 µg/g ± 4.2% (eight replicates). Analysis of the samples was performed by single determination and the concentration of MK-4 used for input in the BBD.

### 4.10. Modelling in JMP

As mentioned, the experimental design and multiple linear regression analysis were performed using the software JMP Pro 16 (SAS Institute, Cary, NC, USA). Briefly described, models were fitted using the Standard Least Squares Personality, resulting in a Fit Least Squares report. First, insignificant effects were removed one at a time until only significant effects (*p*-value < 0.05) remained. The statistical reliability of the models was then evaluated based on the Analysis of Variance (ANOVA), lack of fit (LOF), and Summary of Fit (SOF) output. A model was considered statistically sound if having an ANOVA *p*-value < 0.05 and a LOF *p*-value > 0.05. Model strength, more precisely the ability to describe variation in the design space, was further assessed by the LOF R^2^ and R^2^-adj. values. Preferably, these two should be similar and close to the value 1.0. A summary of the ANOVA, LOF, and SOF for each model is found in supplementary Table A1.

## 5. Conclusions

In this study, *Nannochloropsis oceanica* cultured under nutrient-replete conditions was subjected to different combinations of temperature, light intensity, and salinity to understand their effect on growth and chemical composition. Using response surface methodology, biomass productivity and the content of protein, EPA, and MK-4 were modelled individually. The results from the RSM experiment revealed that biomass productivity and protein content was highest at 27 °C combined with 300–350 µmol·m^−2^·s^−1^. In contrast, lower cultivation temperatures enhanced the production of both EPA and MK-4, with the two peaking at the lowest tested temperature (19 °C). These results highlight a clear tradeoff between biomass accumulation and induction of nutritionally valuable lipids. This could potentially be solved by applying two-stage cultivation, changing temperature and light intensity to first favor growth and later accumulation of EPA and MK-4. Taken together, this study provides valuable information on *N. oceanica*’s cultivational behavior and provides a tool for predicting the nutritional value of *N. oceanica*’s biomass produced under overall replete and non-stressful conditions.

## Figures and Tables

**Table 1:** Box–Behnken design. Coded and actual values for the cultivation variables, followed by the measured and model-predicted results for biomass productivity, protein, eicosapentaenoic acid (EPA), and menaquione-4 (MK-4). Actual values, except for MK-4 (single technical measurement), are the mean of a double determination of one biological replicate (*n* = 1).

Unit	Code	Temperature (°C)	Light Intensity (µmol·m^−2^·s^−1^)	Salinity (% *w*/*v*)	Actual Values				Predicted Values			
					Biomass Prod. (g DM·L^−1^·day^−1^)	Protein (g·g^−1^ DM)	EPA (g·g^−1^ DM)	MK-4 (µg·g^−1^ DM)	Biomass Prod. (g DM·L^−1^·day^−1^)	Protein (g·g^−1^ DM)	EPA (g·g^−1^ DM)	MK-4 (µg·g^−1^ DM)
1	++0	27	360	3.0	0.120	0.566	0.025	83.0	0.135	0.558	0.029	73.8
2	+-0	27	120	3.0	0.081	0.534	0.030	62.4	0.078	0.536	0.029	59.9
3	+0+	27	240	3.7	0.123	0.555	0.025	59.3	0.123	0.559	0.027	66.9
4	+0-	27	240	2.3	0.121	0.557	0.027	62.8	0.123	0.559	0.026	66.9
5	000	23	240	3.0	0.114	0.527	0.033	61.2	0.111	0.543	0.033	61.7
6	000	23	240	3.0	0.115	0.552	0.033	60.8	0.111	0.543	0.033	61.7
7	000	23	240	3.0	0.119	0.554	0.033	52.5	0.111	0.543	0.033	61.7
8	000	23	240	3.0	0.111	0.541	0.034	67.0	0.111	0.543	0.033	61.7
9	0++	23	360	3.7	0.141	0.546	0.033	58.0	0.124	0.542	0.031	59.4
10	0-+	23	120	3.7	0.074	0.513	0.035	66.8	0.066	0.520	0.031	64.1
11	0+-	23	360	2.3	0.117	0.540	0.030	56.9	0.124	0.542	0.030	59.4
12	0--	23	120	2.3	0.059	0.522	0.032	70.7	0.066	0.520	0.030	64.1
13	-+0	19	360	3.0	0.116	0.537	0.038	72.0	0.112	0.546	0.037	66.1
14	--0	19	120	3.0	0.050	0.530	0.038	88.5	0.054	0.523	0.037	89.3
15	-0+	19	240	3.7	0.085	0.550	0.032	49.6	0.099	0.546	0.035	77.7
16	-0-	19	240	2.3	0.099	0.545	0.032	72.5	0.099	0.546	0.034	77.7

**Table 2:** Model precision test of data generated for *Nannochloropsis oceanica*. Model-predicted response means and 95% confidence interval (CI) and actual means ± standard deviations (std) for triplicate validation units (*n* = 3) cultivated at 27 °C, 350 µmol·m^−2^·s^−1^ and 3.0% salinity. Differences in predicted and actual means are given as percentages (%).

Model	Predicted Mean [95% CI]	Actual Mean ± Std	Difference in Means (%)
Biomass productivity (g DM·L^−1^·day^−1^)	0.135 [0.124–0.147]	0.124 ± 0.002	8.5
Protein (g·g^−1^ DM)	0.559 [0.548–0.571]	0.520 ± 0.007	7.2
EPA^1^ (g·g^−1^ DM)	0.029 [0.027–0.031]	0.027 ± 0.004	7.1
MK-4 ^2^ (µg·g^−1^ DM)	73.0 [62.6–83.3]	63.5 ± 12.1	13.9

^1^ EPA: Eicosapentaenoic acid; ^2^ MK-4: menaquinone-4.

**Table 3:** Experimental domain of the Box–Behnken design. Chosen cultivation factors and the three levels, actual and encoded. Zero represents the center value.

Factor	-	0	+
Temperature (°C)	19	23	27
Light intensity (µmol·m^−2^·s^−1^)	120	240	360
Salinity (% *w*/*v*)	2.3	3.0	3.7

## Data Availability

All additional data not presented in this article, including Appendix A, Appendix B and Appendix C will be made available on request.

## Appendix A

**Table A1:** ANOVA, lack of fit, and Summary of Fit values for generated models on biomass productivity, protein content, EPA content, and MK-4 content in *N. oceanica*.

Source	DF	Sum of Squares	Mean Square	F Ratio	*p*-Value	R^2/^R^2^ Adj.
Biomass productivity						
Model	3	0.009	0.003	33.91	<0.0001	
Error	12	0.001	0.000			
C. total	15	0.010				
Lack of Fit	5	0.0005	0.0001	1.32	0.356	
Pure Error	7	0.0005	0.00008			
Total Error	12	0.0010				
0.894/0.868						
Protein content						
Model	4	0.0023	0.0006	8.26	0.0025	
Error	11	0.0008	0.00007			
C. total	15	0.0030				
Lack of Fit	4	0.0002	0.00005	0.71	0.610	
Pure Error	7	0.0005	0.00008			
Total Error	11	0.0008				
0.750/0.660						
EPA content						
Model	3	0.0002	0.00005	11.22	0.0008	
Error	12	0.000005	0.000005			
C. total	15	0.0002				
Lack of Fit	5	0.00004	0.000008	4.57	0.036	
Pure Error	7	0.00001	0.000002			
Total Error	12	0.00005				
0.737/0.62						
MK-4 content						
Model	4	942.34	235.6	5.88	0.0107	
Error	10	400.83	40.1			
C. total	14	1343.17				
Lack of Fit	4	279.73	69.93	3.46	0.086	
Pure Error	6	121.10	20.18			
Total Error	10	400.83				
R^2/^R^2^ Adj.						0.702/0.585

## Appendix B

**Table A2:** Fatty acid composition and content of SFAs, MUFAs, and PUFAs in *N. oceanica* biomass with the lowest (unit 1) and highest (unit 13) measured EPA content. Highlighted are the three most abundant FAs, palmitic acid (16:0), palmitoleic acid (16:1, n-7), and EPA (20:5, n-3). All values in g·g^−1^ DM.

Fatty Acid	Unit 1 (Low EPA)	Unit 13 (High EPA)
10:0	-	-
12:0	-	-
13:0	-	-
14:0	0.012	0.008
14:1	0.000	0.000
15:0	0.001	0.000
16:0	0.033	0.026
16:1 (n-7)	0.041	0.037
16:2 (n-4)	0.000	0.001
16:3 (n-4)	0.000	0.000
17:0	0.001	0.001
17:1	-	-
16:4 (n-3)	0.000	0.000
18:0	0.001	0.000
18:1 (n-9)	0.004	0.004
18:1 (n-7)	0.001	0.000
18:2 (n-6)	0.009	0.007
18:2 (n-4)	0.000	0.000
18:3 (n-6)	-	-
18:3 (n-4)	0.000	0.000
18:3 (n-3)	-	-
18:4 (n-3)	0.000	0.000
18:5 (n-3)	-	-
20:0	0.000	0.000
20:1 (n-9, n-11)	-	-
20:1 (n-7)	-	-
20:2 (n-6)	0.000	0.000
20:3 (n-6)	0.000	0.000
20:4 (n-6)	0.005	0.006
20:3 (n-3)	-	-
20:4 (n-3)	0.000	0.000
20:5 (n-3)	0.025	0.038
22:1 (n-11)	-	-
22:1 (n-9)	-	-
21:5 (n-3)	0.000	0.001
22:2	-	-
22:3	-	-
22:4	-	-
22:5 (n-3)	-	-
22:6 (n-3)	-	-
24:1 (n-9)	-	-
22:0	0.000	0.000
24:0	-	-
SFAs	0.048	0.036
MUFAs	0.046	0.042
PUFAs	0.044	0.054
FA total	0.137	0.132

## Appendix C

**Table A3:** Used cultivation time and final DM content at harvest for the main RSM units and validation units (v1–v3) of *N. oceanica*. Validation units were cultivated at 27 °C, 350 µmol·m^−2^·s^−1^, and 3.0% salinity.

Unit	Code	Temperature (°C)	Light Intensity (µmol·m^−2^·s^−1^)	Salinity (% *w*/*v*)	Cultivation Time (days)	DM at Harvest (g DM·L^−1^)
1	++0	27	360	3.0	6.27	0.750
2	+-0	27	120	3.0	8.86	0.720
3	+0+	27	240	3.7	6.85	0.840
4	+0-	27	240	2.3	6.27	0.760
5	000	23	240	3.0	6.85	0.780
6	000	23	240	3.0	6.85	0.790
7	000	23	240	3.0	5.96	0.710
8	000	23	240	3.0	5.96	0.660
9	0++	23	360	3.7	5.02	0.710
10	0-+	23	120	3.7	9.96	0.735
11	0+-	23	360	2.3	6.85	0.800
12	0--	23	120	2.3	8.89	0.520
13	-+0	19	360	3.0	6.23	0.720
14	--0	19	120	3.0	12.78	0.640
15	-0+	19	240	3.7	7.93	0.675
16	-0-	19	240	2.3	6.23	0.615
v1		27	350	3.0	6.24	0.767
v2		27	350	3.0	5.80	0.725
v3		27	350	3.0	5.80	0.715
